# eHealth literacy level and its predictors among primary school teachers

**DOI:** 10.1186/s12889-025-22799-w

**Published:** 2025-04-23

**Authors:** Mehdi Khancolabi, Zahra Nowrozian, Reza Shahrabadi, Mitra Gharib, Hassan Saadati, Saeed Nowrouzian

**Affiliations:** 1 Department of Health Education and Promotion, School of Public Health, North Khorasan University of Medical Sciences, Bojnurd, Iran; 2 Department of Education, Sabzevar, Razavi Khorasan Iran; 3 Department of Health Education and Public Health, School of Health, Sabzevar University of Medical Sciences, Sabzevar, 0000-0003, 3284-6177 Iran; 4 E-learning in Medical Education Department, School of Medicine, Tehran University of Medical Sciences, Tehran, Iran; 5 Department of Epidemiology, North Khorasan University of Medical Science, Bojnurd, Iran; 6 Computer Department, Sabzevar Branch, Islamic Azad University, Sabzevar, Iran

**Keywords:** eHealth literacy, Teachers, Primary schools

## Abstract

**Background and aim:**

The present study aims to investigate eHealth literacy and its related factors among primary school teachers in Sabzevar city.

**Method:**

The current cross-sectional descriptive analytical study was conducted on 252 primary school teachers in Sabzevar city in 2021. The data collection tool included a three-part questionnaire. The first part included 21 demographic questions, the second part of the questionnaire included 8 questions regarding eHealth literacy, and the third part consisted of 10 questions created by the researcher to complete the standard questionnaire. Data analysis was performed using SPSS software and the statistical significance level was *P* ≤ 0.05.

**Results:**

The mean scores of eHealth literacy of the teachers according to the standard and complete questionnaires were 25.44 ± 5.90 and 60.17 ± 10.17, respectively. There was a statistically significant difference in the eHealth literacy levels of the subjects according to the evaluation of the level of general health, regular physical activity, general knowledge in the field of health, the amount of using the Internet, the use of information related to health through social networks based on both questionnaires (standard/complete). For the standard and complete questionnaires, the multiple correlation coefficient values were 0.48 and 0.52, respectively. The adjustment coefficient value for both questionnaires (R^2^_Adjusted_ = 0.20).

**Conclusion:**

The eHealth literacy of the teachers in this study was not appropriate. The mastery of cyberspace, the level of education, having traditional health literacy were among the most important factors affecting the improvement of teachers’ electronic health literacy.

## Introduction

Electronic literacy (E-literacy) includes six types, including traditional, information, media, health, computer, and scientific literacy [1]. eHealth literacy is the ability to search, find, understand, and evaluate health information from electronic sources, and the knowledge used to address or solve a health problem is considered [2]. eHealth literacy is the level of individual skill and competencies needed to provide, communicate, process, and understand basic health information and services needed to make appropriate health decisions [3]. Those who have relatively high eHealth literacy use more efficient web search strategies and have the ability to identify high-quality health information [4]. Studies in this field have indicated that low health literacy is associated with adverse health effects, harmful health behaviors, and in some cases higher mortality. Many believe that low health literacy actually increases health inequalities [5]. Inadequate health literacy is associated with poor health status, inappropriate use of medications, not following physician’s instructions, poor control of blood sugar and blood pressure, increased prevalence of individual reports of problems caused by poor control, less health knowledge, less participation in treatment decision making, and poor doctor-patient communication [6]. People with low health literacy have less knowledge about their health, they do not receive enough preventive services, the control of chronic diseases is not favorable in them, they have weaker physical and mental health performance, and the rate of using emergency units and hospital services is higher in them [7]. However, those who have adequate eHealth literacy manage important health issues and can make informed health decisions [8].

The results of some studies have indicated that people’s health literacy is relatively low [9], so that the studies conducted in Alborz [10, 11] and Ardabil [12] confirm this issue. Also, some global studies reported different levels of eHealth literacy among people in different communities [13, 14]. Because of the difference in demographic variables, they cannot be compared with each other. In some studies, it has been reported favorably and high [15], and in others studies, a lower percentage of people had a high level of eHealth literacy [16]. Among these, the group of teachers can be particularly important [17]. Increasing the level of health literacy among teachers enhances the learning of health literacy among students. Teachers play a significant role in teaching health education in schools, and their level of health literacy and health competence plays a crucial role in this regard [18]. The age group of 6–11 years is of particular importance, people’s knowledge (parents-teachers) is necessary in order to prevent adverse consequences [19]. In a study conducted on students in European countries, it was shown that 13.3% had a low level of health literacy, and 67.2% had a moderate level of health literacy, indicating a difference in the level of health literacy among students [20]. Problems related to the health of students can be an obstacle to effective learning and make the educational process ineffective. One of the most influential factors affecting the health of students and improving their health is teacher, and any change in the direction of improving the level of health in schools depends on the ability and thinking of teachers [21]. The effectiveness of any program carried out to improve and promote health in schools depends on the level of teachers’ health literacy, but studies have shown that many teachers do not have an adequate level of health literacy [22]. A study reported that only 1% of primary teachers had good information about health and physical and mental health needs of students [23]. Teachers will be able to be effective in improving the health of schools and students if they themselves have adequate health literacy [24]. Studies in the field of assessing the level of eHealth literacy among teachers are very limited. However, the findings of previous studies on the health literacy of teachers and practice teachers have shown that almost 45% of the teachers and practice teachers participating in the study had a limited level of health literacy [25]. Given that no study has been conducted to measure the level of eHealth literacy of teachers in Sabzevar, Iran, the present study aims to investigate eHealth literacy and its related factors among primary school teachers in Sabzevar city in 2021.

## Method

The present cross-sectional descriptive analytical study was conducted with the aim of investigating the eHealth literacy of all primary school teachers in Sabzevar city in 2021. According to the results of the previous study [26], the sample size of the study was determined to be at least 225 people, taking into account the mean of 28.73 (± 5/33), the confidence interval of 95% and the test power of 80%. The participants participated in the research knowingly and by completing the consent form. A list of all primary schools in Sabzevar city was prepared from the educational department. Then, by referring to the schools, finally, 252 teachers were selected for this study using a simple random sampling method. All the subjects participated in this study voluntarily and by explaining the objectives and details of the study, written and informed consent was obtained from them. The participants were also assured of the confidentiality of the information. This study was conducted with the approval of the ethics committee of North Khorasan University of Medical Sciences (ethics code IR.NKUMS.REC.1400.148) and with the cooperation of Sabzevar Education Department in 2021.

A three-part questionnaire was used to collect data. The first part of the questionnaire included 21 demographic questions (such as, age, gender, education, level of skill in using the Internet, underlying disease, concern about health status, compliance with dietary regimens, exercise, history of drug use, browsers, health sites, etc.). Also the questions focused on people’s knowledge and understanding of what health information resources are available on the Internet, where a person can find useful health resources, how to access these resources, how to use the Internet in order to answer health related issues, the ability to evaluate online health information and distinguish low-quality sources from quality sources on the Internet The second part of the questionnaire included questions regarding the assessment of eHealth literacy, including 8 standard questionnaire questions [27] and the third part included 10 researcher-made questions. In a study, Bazm et al. [28] examined the validity and reliability of the Iranian version of the EHealth Literacy Scale [27]. They reported the factor loading of the items from 0.723 to 0.862, which are acceptable values, and Cronbach’s alpha coefficient (alpha = 0.88, *p* < 0.001) and the test-retest coefficient values were also reliable (*r* = 0.96) (*p* < 0.001). The results showed that the items in the translated version were equivalent to the original measure and the Iranian version of the eHealth literacy measure showed good validity and reliability regarding the eHealth literacy test of Iranians.

The researchers of the present study believed that in addition to these 8 questions of the standard questionnaire, other questions related to this topic should be used to cover all aspects of eHealth literacy. Finally, according to the opinion of experts in this field, 10 questions were also added. The first draft of the researcher-made questionnaire was evaluated by three relevant experts and the necessary changes and corrections were applied. For confirming reliability, the researcher-made questionnaire was sent to 10 professors of health education and electronic learning, who were experts in the field of study and the items of clarity, simplicity, and relevance of the questions were evaluated and the overall CVR was equal to 0.93. The CVI of all the questions of the questionnaire was obtained above 0.9 and the overall CVI was equal to 0.9, and finally the validity of the questionnaire was confirmed. To measure the reliability, the questionnaire was sent to 30 teachers. The obtained data were entered into SPSS software and Cronbach’s alpha was calculated 0.77. At this stage, one of the questions was removed. In external reliability, test-retest was conducted on 30 teachers, and the correlation coefficient was 0.95 with *p*-value = 0.001, and finally the reliability of the questionnaire was confirmed.

The responses were based on a 5-point Likert scale from completely disagree [1] to completely agree [5]. The final score of each respondent for the standard questionnaire [27]. The two part was from 8 to 40 and for the third part ranged from 10 to 50. Finally, the score in the whole questionnaire varied from 18 to 90, and a higher score indicated high eHealth literacy.

After collecting the data and performing the necessary controls, the data were entered into SPSS 24 software and analyzed using descriptive statistics, such as percentage, ratio, mean, standard deviation. Suitable statistical tests were also used, such as Chi-square (for qualitative variables), independent t-test (for quantitative variables), and multiple linear regression, with a confidence level of 95%.

## Results

Out of 253 participants in this study, 186 people (73.5%) were females and 67 people (26.5%) were males, and the mean age of the participants was 43.28 ± 6.85. The teaching level of 114 teachers (45.1%) was other than the 6 grades of primary school. In terms of education level, 10 people (0.4%) had an associate degree, 137 people (54.2%) had a bachelor’s degree, 102 people (40.3%) had a master’s degree, and 4 people (1.6%) had Ph.D. degree. Moreover, 166 (65.6%) of the teachers had International Computer Driving License (ICDL) certificate. Further details are provided in Table 1. 

**Table 1:** Demographic variables of the participants

Variable		Frequency	%
Teaching level	1	29	11.5
	2	11	4.3
	3	20	7.9
	4	22	8.7
	5	31	12.3
	6	26	10.3
	Others	114	45.1
Gender	Female	186	73.5
	Male	67	26.5
Education level	Associate degree	10	4.0
	Bachelors’ degree	137	54.2
	Masters’ degree	102	40.3
	PhD	4	1.6
ICDL	Yes	166	65.6
	No	87	34.4

### eHealth literacy

The mean scores of eHealth literacies for the subjects according to the standard and complete questionnaires were 25.44 ± 5.90 and 60.17 ± 10.17, respectively.

### Factors related to eHealth literacy

In relation to demographic variables, the results showed that the mean eHealth literacy scores of the subjects based on the evaluation of the level of general health (P_standard, total_ <0.001), the amount of regular physical activity (P_standard_=0.004, P_standard_ =0.01), the amount of general knowledge in the field of health (P_standard_, _total_ <0.001), the amount of using the Internet to obtain general information in the field of health (P_standard,_ total < 0.001), the use of information related to health through social networks (P_total_=0.008,P_standard_=0.02), and the reliability of information related to health through social networks (P_standard_, _total_ <0.001) were different according to both questionnaires (standard/complete). This difference was significant, and the mean score of eHealth literacy according to the level of the complete questionnaire was also significant (*P* = 0.02). No significant difference was observed in other variables. Further details are provided in Table 2.

**Table 2:** Factors related to eHealth literacy

Variable		Health literacy score according to the standard questionnaire		Health literacy score according to the complete questionnaire	
		Mean	Standard deviation	Mean	Standard deviation
Age group	20–30	28.2308	5.41839	65.6154	10.42863
	30–40	26.5185	4.26408	61.0370	7.76323
	40–50	24.3200	5.37735	59.3800	9.50164
	50–60	25.3865	6.25914	59.8405	10.64229
*P*-value*		0.13		0.22	
Gender	Female	25.3387	6.14934	59.7903	10.44130
	Male	25.7313	5.19493	61.2388	9.39550
*P*-value**		0.64		0.32	
Education level	Less than a bachelor’s degree	25.0204	6.11921	58.9728	10.45076
	More than a bachelor’s degree	26.0283	5.56769	61.8396	9.58485
*P*-value**		0.18		0.02	
ICDL	Yes	25.7816	6.60765	60.1724	10.69036
	No	25.2651	5.51271	60.1747	9.93004
*P*-value**		0.51		0.99	
Underlying disease	Yes	25.4480	5.89401	60.2308	10.32015
	No	25.4063	6.06874	59.7813	9.26573
*P*-value**		0.97		0.80	
History of drug use	Yes	25.4821	5.34957	60.1786	9.08767
	No	25.4315	6.06526	60.1726	10.48715
*P*-value**		0.95		0.99	
Concern about health status	Yes	25.9796	5.44017	60.8027	9.73177
	No	24.6981	6.44563	59.3019	10.74924
*P*-value**		0.08		0.25	
The amount of internet use during the day	Less than 1 h	24.3636	6.08564	58.5455	10.40885
	1 to 2 h	24.6863	5.55874	58.3333	9.59722
	2 to 3 h	26.0625	8.97752	60.7500	12.50866
	More than 3 h	26.2750	5.36713	61.7750	9.80607
*P*-Value*		0.13		0.09	
The tool used to connect to the Internet	Tablet	29.4000	6.14817	69.4000	11.39298
	Smartphone	25.3826	5.98992	60.0348	10.21424
	Workplace computer	25.8889	3.65529	59.0000	8.09321
	Home computer	24.3333	5.22015	59.7778	9.48390
*P*-value*		0.45		0.23	
Assessing the level of public health	Very much	26.9815	5.97343	63.1111	9.88658
	Much	26.7778	5.95711	61.7037	11.31308
	To some extent	25.5000	5.00000	56.0000	10.73934
	Low	23.6667	5.40840	57.1754	9.34893
*P*-value*		< 0.001		< 0.001	
History of disease	More than once	25.9512	7.01410	60.4826	9.76273
	Once	25.4378	5.69977	59.7317	11.96667
	No	23.6364	5.22059	56.1818	10.51492
*P*-value*		0.51		0.38	
Regular physical activity	< 5 times a week	27.4545	4.79160	63.9545	9.34375
	3–4 times a week	25.5333	5.55321	60.8333	9.05617
	Once-twice a week	25.4375	4.93751	59.8667	10.00619
	Once − 3 times a month	25.0690	7.54004	59.5172	11.17593
	Rarely	24.3864	6.37602	57.7045	10.52437
	Never	21.1429	3.80476	54.4286	6.92477
*P*-value*		0.01		0.004	
Healthy diet during 24 h a day	Very low	23.7241	4.71999	57.5862	8.67063
	Low	23.7273	5.74614	56.7273	11.09136
	To some extent	25.0000	7.64337	59.4872	11.03314
	Much	25.7500	5.17283	60.9032	9.80660
	Very much	26.4000	6.60241	61.1600	10.92714
P*P*-value*		0.26		0.35	
The amount of general knowledge in the field of health	Very little	20.0000	5.24087	50.9375	8.93285
	Little	24.3538	5.88634	57.8615	9.87971
	To some extent	27.4301	5.11051	63.9032	8.90294
	Much	28.0000	2.82843	68.5000	4.94975
	Very much	28.6667	5.71017	67.2500	9.04660
*P*-value*		< 0.001		< 0.001	
The amount of using the Internet to obtain general information in the field of health	Once or several a day	27.3333	5.94947	63.5000	9.48236
	Once or several times a week	26.5417	4.89665	62.3438	9.15461
	Once or several times a month	24.6389	6.17196	58.4306	10.12155
	Once or several times a year	20.6129	5.31462	51.7097	9.32450
*P*-value*		< 0.001		< 0.001	
Using information related to health through social networks	Very little	23.5517	5.15881	57.3793	9.50719
	Little	24.6897	5.33254	58.5862	9.79255
	To some extent	25.2945	6.30945	59.6781	10.49265
	Much	26.7436	4.79977	63.1026	9.09539
	Very much	30.2000	4.46716	68.7000	6.48160
*P*-value*		0.02		0.008	
Reliability of information related to health through social networks	Very little	25.2692	4.98444	59.5385	9.50465
	Little	22.2200	5.91536	55.1600	10.97745
	To some extent	25.7792	5.80539	60.6753	9.56975
	Much	29.9524	2.51945	68.0000	7.07814
	Very much	35.0000	4.24264	73.0000	0.00000
*P*-value*		< 0.001		< 0.001	

* ANOVA test results

** Independent t-test results

## Factors predicting eHealth literacy

Multiple linear regression analysis was used to investigate the predictive factors of eHealth literacy. Multiple correlation coefficient values for the standard and complete questionnaires were 0.48 and 0.52, respectively. The adjustment coefficient value for both questionnaires (R2Adjusted) was also 0.20, which indicates that the predictor variables of the model were able to predict nearly 20% of the variance of eHealth literacy. The result of the ANOVA, evaluating the significance of the whole model, showed that the model is significant (F_standard_=6.79, F_Total_=7.93 and *P* < 0.001). The results of the multiple linear regression test (Table 4) according to both questionnaires show that the variables of concern about health status (*P* = 0.02), the amount of using the Internet during the day (*P* = 0.02), the amount of regular physical activity (*P* < 0.001), the amount of general knowledge in the field of health (*P* = 0.001), and the use of information related to health through social networks (*P* = 0.05) were significant predictors for eHealth literacy. The standardized regression coefficients for these variables were also positive, indicating the positive effect and prediction of eHealth literacy by the mentioned variables. Further details are provided in Table 3.

**Table 3:** Determining predictors of eHealth literacy based on multiple linear regression analysis

Variable	Standard questionnaire					Complete questionnaire				
	Unstandardized coefficients		Standardized coefficients (β)	t-statistic (*t*-value)	*p*-value	Unstandardized coefficients		Standardized coefficients (β)	t-statistic (*t*-value)	*p*-value
	Coefficient (β)	Standard error				Coefficient (β)	Standard error			
Age group	0.49	0.51	0.06	0.96	0.33	-0.17	0.86	-0.01	-0.19	0.84
Gender	0.53	0.68	0.04	0.77	0.44	1.95	1.16	0.09	1.68	0.09
Education level	0.15	0.16	0.06	0.96	0.34	0.46	0.27	0.09	1.69	0.09
Teaching level	0.35	0.72	0.03	0.48	0.63	-0.21	1.21	-0.01	-0.17	0.86
Concern about health status	0.71	0.36	0.11	1.96	0.05	1.35	0.61	0.13	2.22	0.02
The amount of using the Internet during the day	1.2720	0.53	0.15	2.37	0.02	2.02	0.91	0.14	2.23	0.02
Assessing the level of public health	-0.31	0.24	-0.07	-1.28	0.20	-0.38	0.41	-0.05	-0.91	0.36
The amount of regular physical activity	1.65	0.50	0.20	3.26	0.001	3.28	0.85	0.23	3.85	< 0.001
The amount of general knowledge in the field of health	-1.27	0.39	-0.20	-3.27	0.001	-2.29	0.66	-0.21	-3.48	0.001
The amount using the Internet to obtain general information in the field of health	-0.29	0.51	-0.04	-0.57	0.57	-0.22	0.85	-0.02	-0.26	0.79
Using information related to health through social networks	1.38	0.59	0.18	2.33	0.02	1.98	1.00	0.15	1.98	0.05
Reliability of information related to health through social networks	0.49	0.51	0.06	0.96	0.33	-0.16	0.86	-0.01	-0.19	0.84

## Discussion

Although eLearning has been increasingly developed in recent years in various dimensions and many organizations have shown a desire to discover new methods, many organizations have not understood its advantages well. Infrastructure for increasing eHealth literacy is an approach that should be considered by many organizations. To this end, this descriptive study aims to investigate the eHealth literacy of primary school teachers in Sabzevar city in 2021.

In the current study, the mean and standard deviation score of eHealth literacy of the teachers according to the standard questionnaire was about 25.44 ± 5.90, which is slightly lower than the standard score of 32 and is not desirable [29]. In some studies [15, 27] the level of eHealth literacy in the participants were higher than the value obtained in the present study. This difference may be due to variations in their communities as well as differences in their demographic variables. It is due to the fact that the education level and age are two effective factors in eHealth literacy [30]. In line with the study of Dashti et al. [30], in this study it was also found that people with higher literacy rate and younger age have higher health literacy compared to other groups. It should be noted that due to the smaller range of education level, the age criterion had a greater effect on the level of eHealth literacy, so that the level of health literacy in the age group of 20–30 years (28.23 ± 5.41) had a smaller difference compared to the value obtained in the study of Dashti et al. This result is consistent with the study by Huang [31], which showed a relationship between age and the level of e-health literacy among teachers, such that teachers of older age had lower functional eHealth literacy. This finding can be attributed to the higher level of access of this group to virtual space. In further comparison with previous studies, it was found that in studies on university student groups, compared to the teachers studied in the present study, the level of health literacy obtained higher scores in both standard and complete questionnaires. In the study by Haerian et al. [32], the mean score of eHealth literacy of students was 76.88, and in the study of Mahmoudi et al., based on the standard questionnaire, the level of health literacy was average [29].

The evaluations in this study showed that increasing the education level, especially at relatively young ages, improved people’s health literacy due to the greater access of these groups to the Internet.

The amount of using the Internet and the type of tool used to connect to the Internet are other factors affecting the level of eHealth literacy. Therefore, people who connect to the Internet daily and with the help of more convenient tools, such as smartphones or tablets had a higher level of health literacy. It is due to the fact that since people with tablets and the most hours of Internet use have the highest health literacy score compared to other groups. These findings are consistent with the study by Isazadeh et al. [33], in which they examined the eHealth literacy of patients referring to a military hospital. It was pointed out that the amount of Internet use improved health literacy [33], due to getting more information through social networks and websites. Considering that using social networks through smartphones is much easier than home and office computers, the level of health literacy improvement is higher in the groups owning tablets and smartphones.

The gender of the teachers had an insignificant effect on the findings of this study, so that in the standard questionnaire, males scored slightly higher and in the complete questionnaire, males scored relatively higher. This result is not in line with the studies of Isazadeh et al. and Richtering et al. [15, 33, 34]. However, it was in good agreement with the study of Norman et al. in 2006. In the study of Norman et al., males had higher eHealth literacy [29]. This result could be due to to the higher access of males to electronic tools.

The results also revealed that according to the standard questionnaire, the mean score of eHealth literacy score of people with diseases and a history of drug use was higher than healthy people. This finding is in contrast with the findings reported in the studies of Haerian et al. regarding oral and dental health, Raeisi et al., and Wolf et al. [30, 35, 36]. Although these disagreements are due to the differences in the demographic variables of the participants, it is expected that people who have health problems have benefited more from electronic resources to deal with their problems.

According to the results, it can be concluded that having dietary regimens and regular exercise as well as having general knowledge led to higher scores among the teachers. This study shows the mutual influence of health literacy and health behavior of people [37, 38].

The studied teachers mentioned that they have the ability to evaluate and recognize the correctness of health-related information and use them in their daily lives. This finding shows that they have the ability to identify accurate and quality sources that have had positive results in their lives. This result is not consistent with the study of Rasouli et al., in which they stated that the people referring to Tehran military hospitals did not have the ability to identify accurate health-related sources and as a result, did not trust the information available on the Internet [33]. This difference can be due to the amount of false information available on the internet and also the low level of education in the group of military patients compared to teachers. This finding shows that the education level not only increases health literacy, but also affects the finding of accurate and reliable sources.

Given the significant impact of teachers’ health literacy levels on students’ learning of health literacy during the education they provide, it is essential to incorporate educational content aimed at enhancing teachers’ eHealth literacy in to the curriculum and teacher training programs so that they can be more competent to play role in transferring this knowledge to the students.

One of the limitations of this study is using self-measurement and, in some cases, it is possible that the responses do not reflect real health literacy. Another limitation is the lack of functional, critical, and interactive dimensions in the eHealth literacy measure, leading to differences with other similar studies. Moreover, some teachers did not participate in the study due to various reasons, such as cultural and family, and environmental conditions. It would be valuable for future studies that use mixed methods for exploring cross-cultural aspects of eHealth literacy that could provide deeper insights.

## Conclusion

In general, the level of eHealth literacy of the teachers in this study was not optimal. On the other hand, due to the increasing growth of social networks and the Internet, health literacy-related information is increasingly published. Therefore, dealing with eHealth literacy in order to accurately process information with minimum cost is becoming more noticeable. The study results indicated that familiarity with virtual space, education level, and traditional health literacy were among the most important factors affecting the promotion of eHealth literacy of the teachers. By better identifying the factors influencing the level of eHealth literacy among teachers, integrated educational programs can be designed and incorporated to enhance teachers’ health literacy during teacher training courses as well as in retraining programs.

## Data Availability

Data related to the current study are available from the corresponding author upon request.
